# LGR5 overexpression confers poor relapse-free survival in breast cancer patients

**DOI:** 10.1186/s12885-018-4018-1

**Published:** 2018-02-22

**Authors:** Ming-Feng Hou, Po-Ming Chen, Pei-Yi Chu

**Affiliations:** 1 Department of Surgery, College of Medicine, Kaohsiung Medical University, Kaohsiung, Taiwan; 2 Department of Surgery, Kaohsiung Municipal Hsiao Kang Hospital, Kaohsiung, Taiwan; 3 Division of Breast Surgery, Kaohsiung Medical University Hospital, Kaohsiung, Taiwan; 4 Taiwan Agricultural Chemicals and Toxic Substances Research Institute, Council of Agriculture, Taichung, Taiwan; 5 Department of Pathology, Show Chwan Memorial Hospital, No. 542, Sec. 1, Chung-Shang Road, Changhua City, Changhua County 50008 Taiwan, Republic of China; 6 School of Medicine, College of Medicine, Fu Jen Catholic University, New Taipei City, Taiwan; 7 National Institute of Cancer Research, National Health Research Institutes, Tainan, Taiwan

**Keywords:** Breast cancer, Cancer stem cell, LGR5

## Abstract

**Background:**

Cancer stem cells (CSCs) are believed to promote the malignant transformation of breast cancer via multiple signaling pathways, including the Wnt/β-catenin pathway. Leucine-rich repeat-containing G protein-coupled receptor 5 (LGR5) has been identified as a CSC-associated Wnt-regulated target gene, but its clinical significance in the context of breast cancer remains elusive. Therefore, the purpose of this study was to investigate the clinical significance of the LGR5-β-catenin axis in breast cancer.

**Methods:**

Breast cancer tissue blocks from 126 patients were used to construct a tissue microarray (TMA). Histopathological and clinical data including age; tumor size; estrogen receptor (ER), progesterone receptor (PR), and human epidermal growth factor receptor 2 (HER2) level; tumor grade; lymph node (LN) status; and survival were obtained from the cancer registry database and patients’ medical records. Tissue on the breast TMA was scored for LGR5 and β-catenin expression using semi-quantitative immunohistochemical (IHC) staining. We also analyzed LGR5 expression in cellular datasets available through ONCOMINE, a web-based cancer microarray database.

**Results:**

Immunohistochemical staining revealed that 58 tumors (46%) exhibited high LGR5 expression, whereas 56 tumors (47%) displayed high β-catenin expression. High levels of LGR5 expression were significantly associated with tumor size (*p* = 0.002), LN metastasis status (*p* = 0.044), and triple-negative breast cancer (*p* = 0.029), consistent with our findings from the ONCOMINE database. In addition, we also found that β-catenin -expressing breast cancers were positive correlated with HER2 overexpression. Finally, with respect to clinical outcomes, patients with high levels of LGR5-β-catenin axis expression exhibited poorer relapse-free survival (RFS) compared to patients with low levels of LGR5-β-catenin axis expression (*p* = 0.027).

**Conclusion:**

LGR5 overexpression was significantly associated with high T stage and LN metastasis status. High LGR5 expression was also associated with reduced RFS, indicating that LGR5 may represent a promising prognostic marker for breast cancer patients.

## Background

Breast cancer is the most frequently diagnosed cancer type and the second-leading cause of cancer-related deaths among women worldwide [1]. Breast cancer in Asia, including Taiwan, is characterized by early tumor onset, and thus exhibits a relatively younger median age at diagnosis than in Western populations [2]. Breast cancer is a heterogeneous disease consisting of several molecular subtypes. Indeed, estrogen receptor (ER) and progesterone receptor (PR) expression, as well as human epidermal growth factor receptor 2 (HER2/Neu) amplification, are associated with distinct subtypes, with prognostic and therapeutic implications [3]. In particular, triple-negative breast cancers (TNBCs), which account for approximately 10–15% of all breast cancer cases, are negative for ER, PR, and HER2, and exhibit poor prognoses relative to other breast cancer subtypes [3].

Breast cancer originates from the epithelial cells of the mammary gland, and, in some cases, is thought to arise from putative cancer stem cells (CSCs) [4]. CSCs are believed to promote the malignant transformation of many cancer types, including breast cancer, in part via activation of the Wnt/β-catenin pathway. Moreover, the CSC-associated marker leucine-rich repeat-containing G protein-coupled receptor 5 (LGR5) has been previously shown to promote breast cancer progression and CSC maintenance, in part through activation of Wnt/β-catenin signaling [5]. However, little is known about the associations between LGR5 expression and breast cancer clinicopathological features.

In the present study, we first examined cellular LGR5 expression levels using datasets available through ONCOMINE, a web-based cancer microarray database. Subsequently, we constructed a tissue microarray (TMA) using specimens from 126 breast cancer patients, and performed semi-quantitative immunohistochemistry (IHC) for LGR5 and β-catenin. We further investigated the associations between LGR5 and β-catenin expression level and 5-year relapse-free survival (RFS) in breast cancer patients.

## Methods

### Patients

This study was approved by the ethics committee of the Institutional Review Board of Kaohsiung Medical University Hospital (KMUH-IRB-2013033). Informed consent was obtained from all participants in accordance with the Declaration of Helsinki. Primary tumor tissues were obtained from 126 breast cancer patients undergoing surgical resection at Kaohsiung Medical University Hospital between 2004 and 2008. Patient characteristics and clinical outcomes were collected until death, censorship, or loss to follow-up. Breast tumor tissue cores were collected from each patient, and used to construct a TMA. Clinical parameters and overall survival data were obtained from patients’ medical records. Patients who had not experienced disease recurrence or metastasis at the end of the study were censored at the date of the last follow up.

### Immunohistochemistry and scoring

Immunohistochemistry was used to detect LGR5 and β-catenin protein expression. The anti-LGR5 antibody (orb137136) was purchased from Biorbyt (Cambridge, UK) and the anti-β-catenin antibody (610154) was purchased from BD Transduction Laboratories™ (Franklin Lakes, NJ, USA). Formalin-fixed paraffin-embedded breast cancer tissue sections (4-μm) on poly-1-lysine-coated slides were deparaffinized with xylenes and rinsed with 10 mM Tris-HCl (pH 7.4) and 150 mM sodium chloride. Endogenous peroxidase was quenched with 3% hydrogen peroxide in methanol. The slides were then placed in 10 mM citrate buffer (pH 6.0) at 100 °C for 20 min in a pressurized heating chamber. Slides were then incubated with LGR5 (1:100) and β-catenin (1:300) antibodies for 1 h at room temperature, and washed three times with phosphate-buffered saline (PBS). Bound antibodies were detected using the EnVision Detection Systems Peroxidase/DAB, Rabbit/Mouse kit (Dako, Glostrup, Denmark) and counterstained with hematoxylin. Finally, the slides were photographed with a BX50 microscope (OLYMPUS, Japan). Colonic adenocarcinoma was used as positive control for LGR5 and β-catenin expression. Negative controls were obtained by performing all of the IHC steps, excluding addition of the primary antibody.

The signal intensities of the slides were evaluated by two board-certified pathologists. Immunostaining score (range: 0, 2–8) was defined as proportion score + intensity score in accordance with a previous report (proportion score: 0 = 0/100, 1 = 1/100~ 1/10, 2 = 1/10~ 1/3, 3 = 1/3~ 2/3, 4 = 2/3~ 1, and 5 = 100/100; intensity score: 0 = negative, 1 = weak, 2 = intermediate, and 3 = strong) [6]. The median IHC staining score (6) was used as the cut-off point for the dichotomization of both LGR5 and β-catenin; Scores greater or equal 6 (≥ 6) were defined as indicating “high” immunostaining, while scores of less than 6 were considered to indicate “low” immunostaining.

### Statistical analysis

Chi-square analysis was conducted using SPSS software (Version 18.0 SPSS Inc., Chicago, IL, USA). Statistical differences in survival data were analyzed using the log-rank test. Survival curves were plotted using the Kaplan-Meier method and SPSS software. A *p*-value of less than 0.05 was considered to indicate statistical significance.

## Results

### Tumor size and lymph node metastasis are associated with high LGR5 expression

**Fig. 1:**
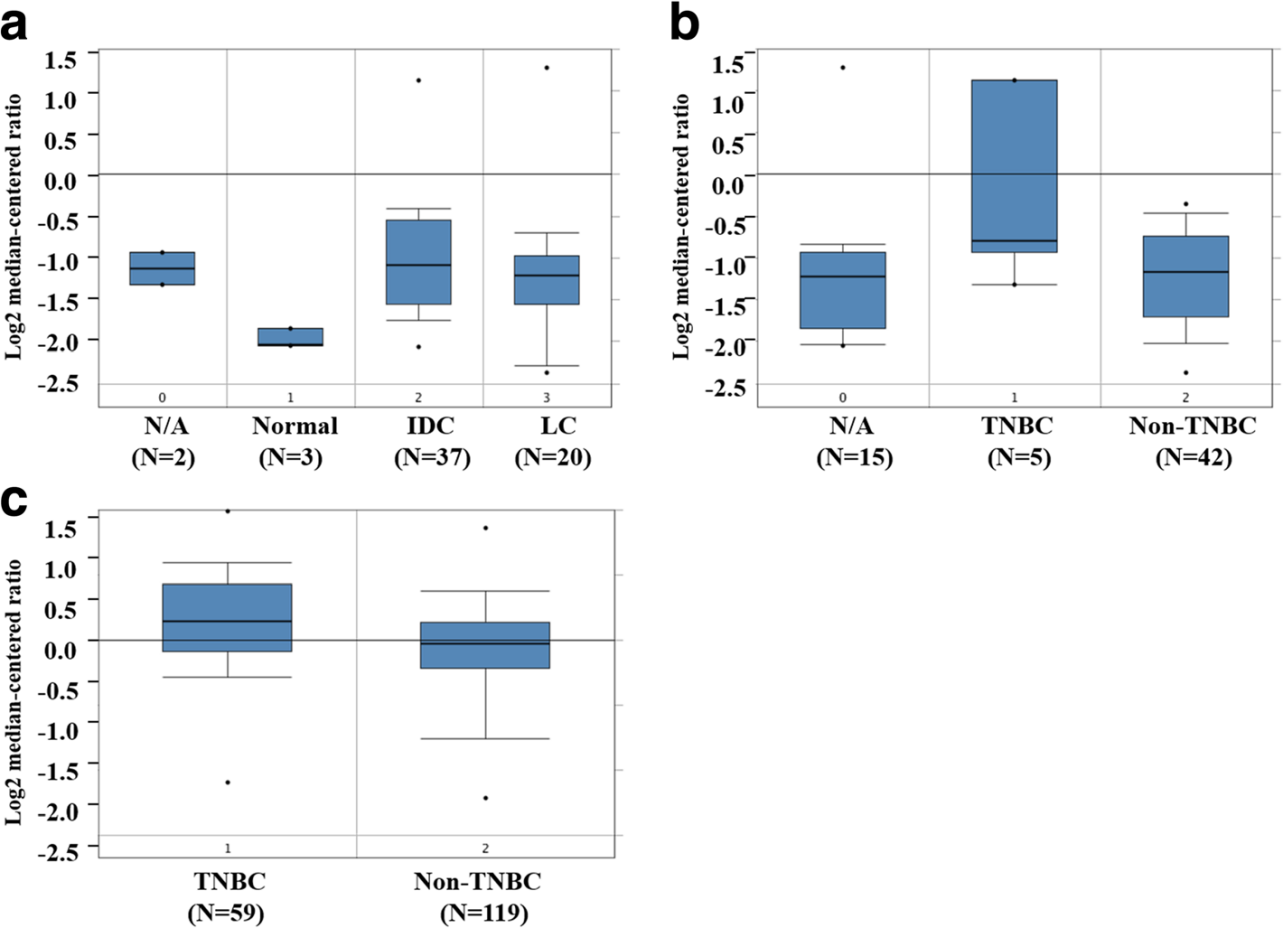
LGR5 expression in ONCOMINE breast tissue datasets. **a** LGR5 expression stratified by tissue type (cancer vs. normal) in the Zhao breast dataset. **b-c** LGR5 expression stratified by triple negative status in the Zhao (**b**) and Tabchy (**c**) breast datasets. TNBC: triple-negative breast cancer; N/A: not available

**Fig. 2:**
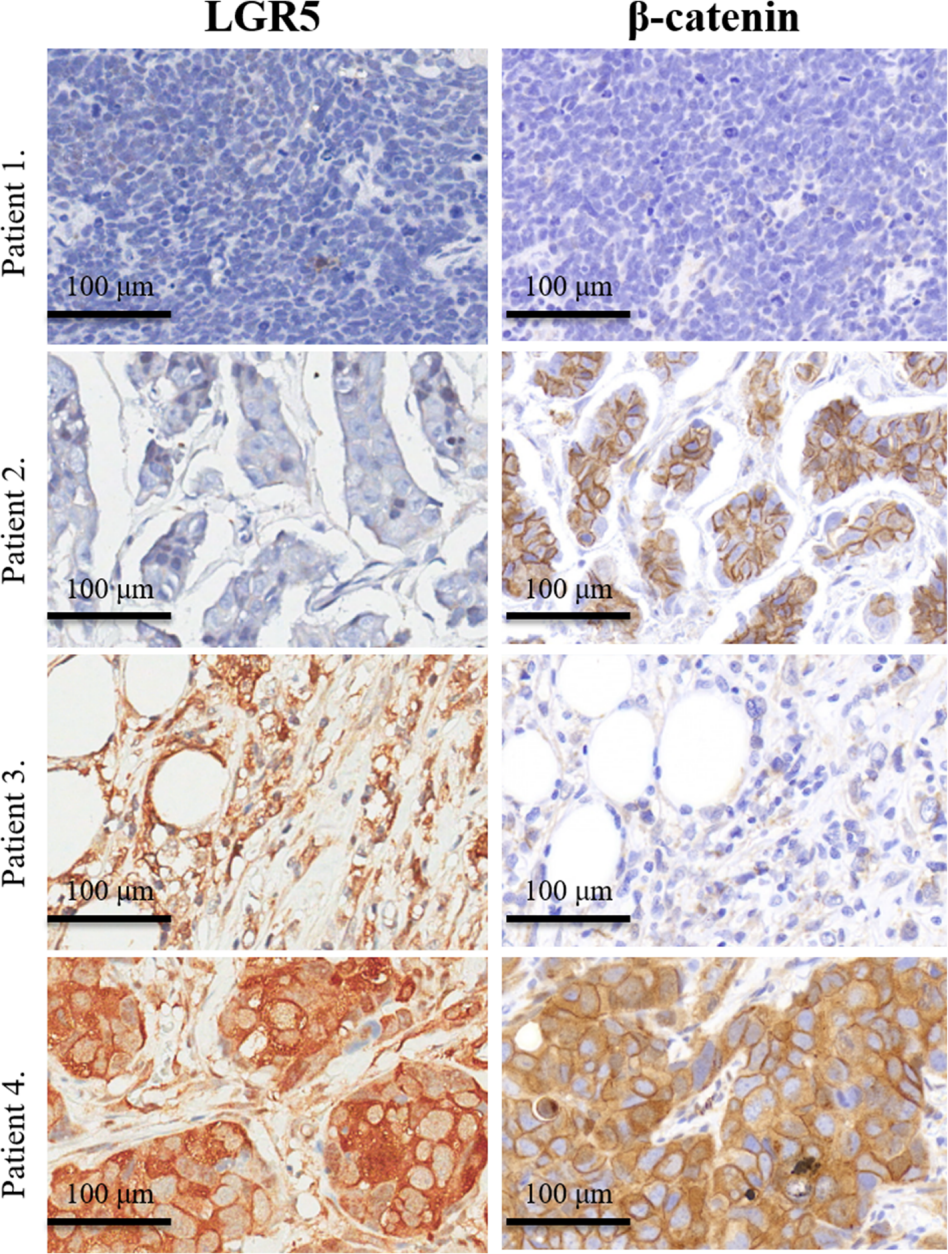
Representative immunohistochemical staining of LGR5 and β-catenin expression. Patient 1: low LGR5/low β-catenin expression. Patient 2: low LGR5/high β-catenin expression. Patient 3: high LGR5/low β-catenin expression. Patient 4: high LGR5/high β-catenin expression

We analyzed LGR5 expression in cellular datasets available through ONCOMINE (http://www.oncomine.org/), an online collection of microarrays. Using the Zhao breast microarray dataset [7], we observed that LGR5 expression was lower in normal breast compared to breast cancer tissue (17 invasive ductal carcinomas and 20 lobular carcinomas) (Fig. 1a). Additionally, mining of the Zhao and Tabchy breast microarray datasets [7, 8] revealed that TNBCs exhibited higher levels of LGR5 expression than non-TNBCs (Fig. 1b and c). These results imply that LGR5 expression is associated with cancer progression. We next used specimens obtained from 126 breast cancer patients to investigate the clinical importance of LGR5 expression. Tumor LGR5 expression was detected via IHC, and representative results are shown in Fig. 2. Of the 126 tumors included in this study, 121 (96.0%) were invasive ductal carcinomas (Table 1). We then determined whether LGR5 expression was associated with clinicopathological tumor features, including age, tumor grade, tumor size, lymph node (LN) status, ER status, PR status, and HER2 status. We found that tumor size ≥ 2 cm, LN metastasis, and TNBC were associated with high levels of LGR5 expression (*p* = 0.002, *p* = 0.044 and *p* = 0.029, respectively; Table 1). These results indicate that LGR5 expression levels were correlated with a degree of tumor malignancy.

**Table 1:** Relationship of clinical parameters with LGR5 expression in 126 breast carcinoma patients

		LGR5 IHC		*p-*value		β-catenin IHC		*p-*value
Variables	N	Low (%)	High (%)		N	Low (%)	High (%)	
Total	126	68 (54)	58 (46)		119	63 (53)	56 (47)	
Differentiation grade								
I	3	3 (100)	0 (0)	0.072	2	2 (100)	0 (0)	0.377
II	93	53 (57)	40 (43)		88	45 (51)	43 (49)	
III	30	12 (41)	18 (59)		29	16 (55)	13 (45)	
Age group								
< = 35	7	5 (71)	2 (29)	0.775	7	4 (57)	3 (43)	0.419
36–45	28	15 (54)	13 (46)		28	15 (54)	13 (46)	
46–55	47	27 (57)	20 (43)		42	23 (55)	19 (45)	
56–65	27	13 (48)	14 (52)		26	10 (38)	16 (62)	
> 65	17	8 (47)	9 (53)		16	11 (69)	5 (31)	
Tumor size (cm)								
< 2	44	33 (75)	11 (25)	0.002	41	21 (51)	20 (49)	0.910
2–5	70	40 (43)	30 (57)		66	35 (53)	31 (47)	
> 5	12	5 (39)	7 (61)		12	7 (58)	5 (42)	
LN metastasis								
Negative	75	46 (61)	29 (39)	0.044	70	36 (51)	34 (49)	0.693
Positive	51	22 (43)	29 (57)		49	27 (55)	22 (45)	
Histopathological feature								
Invasive ductal carcinoma	121	65 (54)	56 (46)	0.830	114	60 (53)	54 (47)	0.178
Invasive mucinous carcinoma	1	1 (100)	0 (0)		1	1 (100)	0 (0)	
Invasive lobular carcinoma	2	1 (50)	1 (50)		2	2 (100)	0 (0)	
Metaplastic carcinoma	2	1 (50)	1 (50)		2	0 (0)	2 (100)	
ER status								
Negative	49	22 (45)	27 (55)	0.103	46	22 (48)	24 (52)	0.375
Positive	77	46 (60)	31 (40)		73	41 (56)	32 (44)	
PR status								
Negative	63	31 (49)	32 (51)	0.284	59	30 (51)	29 (49)	0.650
Positive	63	37 (59)	26 (41)		60	33 (55)	27 (45)	
HER2 status								
Negative	59	27 (46)	32 (54)	0.097	55	35 (64)	20 (36)	0.037
Positive	66	40 (60)	26 (40)		63	28 (44)	35 (56)	
Missing	1				1			
Molecular subtype								
Non-TNBC	109	63 (57)	46 (43)	0.029	102	57 (56)	45 (44)	0.115
TNBC	17	5 (37)	12 (63)		17	6 (35)	11 (65)	

### Association between LGR5 and β-catenin in TNBC

LGR5 in adult breast CSCs may maintain stemness by activating Wnt/β-catenin signaling [5, 9]. Accordingly, blockade of the Wnt/β-catenin signaling pathway reduced metastatic potential by altering CSC activity in a mouse model of breast cancer [10]. Therefore, we used our TMAs to investigate β-catenin staining levels in human breast tumors. Our results indicated that β-catenin expression was positively correlated with HER2 status (*p* = 0.037), but was not associated with age, tumor differentiation status, tumor size, LN metastasis, histopathological features, ER status, and PR status (Table 1). We also examined whether β-catenin was differentially associated with LGR5 as a function of breast cancer molecular subtype. As shown in Table 2, β-catenin and LGR5 expression were positively correlated among TNBCs (*p* = 0.013, Table 2).

**Table 2:** Association between LGR5 and β-catenin protein expression in breast cancer by stratifying molecular subtype

		β-catenin IHC		
	N	Low (%)	High (%)	*p-*value
All patients, LGR5 IHC				
Low	62	33 (53)	29 (47)	0.948
High	57	30 (53)	27 (47)	
Non-TNBC, LGR5 IHC				
Low	57	29 (51)	28 (49)	0.252
High	45	28 (62)	17 (38)	
TNBC, LGR5 IHC				
Low	5	4 (80)	1 (20)	0.013
High	12	2 (17)	10 (83)	

### LGR5 expression is associated with shorter RFS in non-TNBC breast carcinoma patients

**Fig. 3:**
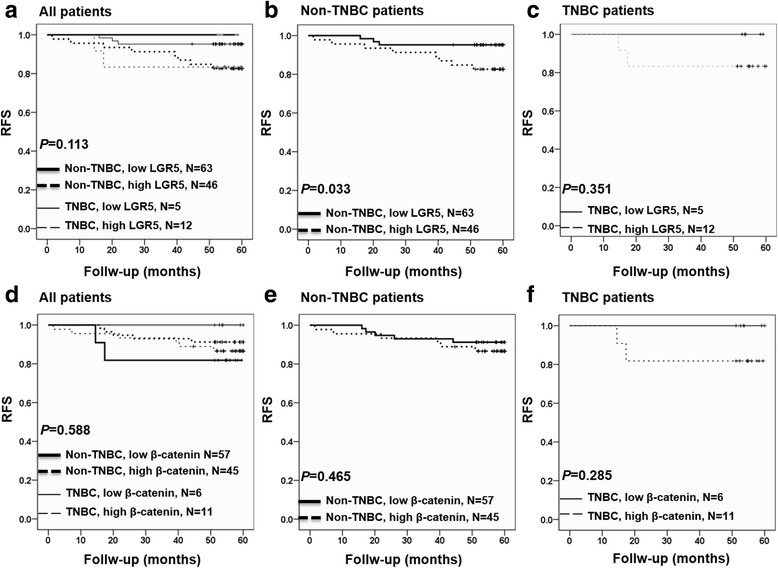
Kaplan-Meier analysis of the association of LGR5 and β-catenin expression with RFS in breast cancer patients. **a** LGR5 expression level in all patients. **b** LGR5 expression level in non-TNBC patients. **c** LGR5 expression level in TNBC patients. **d** β-catenin expression level in all patients. **e** β-catenin expression level in non-TNBC patients. **f** β-catenin expression level in TNBC patients. TNBC: triple-negative breast cancer

We next investigated associations between LGR5 expression, breast cancer recurrence, and patient mortality. As shown in Table 3, 13 patients (9%) experienced recurrence and/or death. A Kaplan-Meier analysis showed that, in the non-TNBC group, patients with high levels of tumor LGR5 expression exhibited significantly shorter RFS periods compared to patients with low levels of LGR5 expression (*p* = 0.033, Fig. 3). Additionally, 8 of 46 non-TNBC patients (13%) with high LGR5 expression experienced recurrence and/or death (Table 3).

**Table 3:** Association between LGR5 expression and 5-year RFS percentage by stratifying molecular subtype

Protein expression	Molecular subtype	Total	Percent	First event (Recurrence and/or death)	Percent
LGR5		126		13	9.0
Low	Non-TNBC	63	50.0	3	7.9
Low	TNBC	5	5.7	0	0.0
High	Non-TNBC	46	36.1	8	13.0
High	TNBC	12	8.2	2	16.7
β-catenin		119		13	10.9
Low	Non-TNBC	57	47.9	5	8.8
Low	TNBC	6	5.0	0	0.0
High	Non-TNBC	45	37.8	6	13.3
High	TNBC	11	9.2	2	18.2
LGR5/ β-catenin		102		11	10.8
Low/ Low	Non-TNBC	29	50.0	2	6.9
Low/ High	Non-TNBC	28	5.7	1	3.6
High/ Low	Non-TNBC	28	36.1	3	10.7
High/ High	Non-TNBC	17	8.2	5	29.4
LGR5/ β-catenin		17		2	11.8
Low/ Low	TNBC	4	23.5	0	0.0
Low/ High	TNBC	1	5.9	0	0.0
High/ Low	TNBC	2	11.8	0	0.0
High/ High	TNBC	10	58.8	2	20.0

### High simultaneous expression of both LGR5 and β-catenin is associated with poor RFS in breast carcinoma patients

**Fig. 4:**
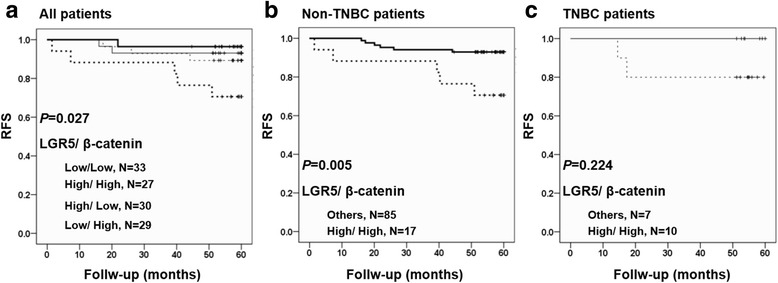
Kaplan-Meier analysis of the association of combined LGR5 and β-catenin expression with RFS in breast cancer patients. **a** All patients. **b** Non-TNBC patients. **c** TNBC patients. TNBC: triple-negative breast cancer

Patients whose tumors exhibited high β-catenin levels, but not high LGR5 levels, did not display significantly shorter RFS periods compared to patients with low levels of tumor β-catenin expression (Fig. 3d-f). However, patients with high tumor β-catenin expression were more likely to experience recurrence and/or death (Table 3). Moreover, Kaplan-Meier analysis of LGR5 together with β-catenin expression revealed that breast cancer patients with high simultaneous expression of tumor LGR5 and β-catenin expression had the worst prognosis (*p* = 0.027, Fig. 4).

## Discussion

This study showed that high levels of LGR5 expression were significantly associated with tumor size ≥ 2 cm and LN metastasis in breast cancer patients (Table 1), and that poorly differentiated tumors exhibited a trend toward higher LGR5 expression (*p* = 0.072, Table 1). Using a Kaplan-Meier analysis, we also found that high levels of LGR5 expression were significantly associated with shorter RFS in non-TNBC patients. This result was not observed in the TNBC group, potentially due to the limited sample size (Fig. 3). Therefore, these findings indicate that LGR5 is a promising marker of poor prognosis, particularly in non-TNBC patients.

LGR5 has been gradually accepted as the most reliable marker for colorectal, breast, pancreatic, and gastric CSCs [5, 11–15]. In fact, studies of the eye, brain, hair follicle, mammary gland, stomach, and reproductive organs have demonstrated that LGR5 expression is increased in rare stem cells, and that these cells may ultimately become CSCs [5]. Recent studies have explored the function of LGR5 in various cancer types. For example, in skin squamous cell carcinoma, LGR5 modulates Wnt/β-catenin signaling by interacting with and cointernalizing Wnt receptors and delaying endosome degradation [15]. In addition, interconversion of LGR5-positive CSCs to LGR5-negative cells has been shown to facilitate drug resistance in colon cancer [15]. However, the mechanism by which LGR5 may promote breast cancer is not fully understood. Therefore, given that β-catenin was positively correlated with HER2 status in the present study (Table 1), we suggest that the LGR5-β-catenin axis is responsible for breast cancer progression.

Overall, our clinical data showed that breast cancer patients with high levels of tumor LGR5 expression have shorter RFS compared to those with low levels of tumor LGR5 expression. However, given that we used an online dataset to analyze LGR5 expression in various cell lines, further pharmaceutical and genomic studies are crucial. Nevertheless, taken together, our study indicates that the development of drugs that inhibit LGR5 expression will be an important strategy for breast cancer treatment.

## Conclusion

Our study revealed that breast cancer patients with high tumor LGR5-β-catenin axis expression have poorer clinical outcomes than those with low tumor LGR5-β-catenin axis expression. Additionally, analysis of ONCOMINE data revealed that LGR5 expression was more highly expressed in TNBC compared to non-TNBC cases. Therefore, LGR5 is a likely therapeutic target in breast cancer patients.

## Abbreviations

CSC: Cancer stem cell
ER: Estrogen receptor
HER2: Human epidermal growth factor receptor 2
IHC: Immunohistochemistry
LN: Lymph node
OS: Overall survival
PR: Progesterone receptor
RFS: Relapse-free survival
TMA: Tissue microarray
TNBC: Triple negative breast cancer
